# The Articulated Oral Airway as an aid to mask ventilation: a prospective, randomized, interventional, non-inferiority study

**DOI:** 10.1186/s12871-021-01315-8

**Published:** 2021-03-29

**Authors:** Ron O. Abrons, Patrick Ten Eyck, Isaac D. Sheffield

**Affiliations:** 1 Associate Professor, Department of Anesthesia, University of Iowa Carver College of Medicine, Iowa City, IA USA; 2 Assistant Director for Biostatistics and Research Design, Institute for Clinical and Translational Science, University of Iowa, Iowa City, IA USA; 3 Resident in Anesthesiology, Department of Anesthesia, University of Iowa Carver College of Medicine, Iowa City, IA USA

**Keywords:** Mask ventilation, Morbid obesity, Oral airway

## Abstract

**Background:**

Oropharyngeal airways are used both to facilitate airway patency during mask ventilation as well as conduits for flexible scope intubation, though none excel at both. A novel device, the Articulated Oral Airway (AOA), is designed to facilitate flexible scope intubation by active displacement of the tongue. Whether this active tongue displacement also facilitates mask ventilation, thus adding dual functionality, is unknown. This study compared the AOA to the Guedel Oral Airway (GOA) in regards to efficacy of mask ventilation of patients with factors predictive of difficult mask ventilation. The hypothesis was that the AOA would be non-inferior to the GOA in terms of expiratory tidal volumes by a margin of 1 ml/kg, thus demonstrating dual functionality.

**Methods:**

In this randomized controlled clinical trial, fifty-eight patients with factors predictive of difficult mask ventilation were mask ventilated with both the GOA and the AOA. Video of the anesthetic monitors were evaluated by a blinded member of the research team, noting inspiratory and expiratory tidal volumes and expiratory CO2 waveforms.

**Results:**

The AOA was found to be non-inferior to the GOA at a margin of 1 ml/kg with a mean weight-standardized expiratory tidal measurement 0.45 ml/kg lower (CI: 0.34–0.57) and inspiratory tidal measurement 0.109 lower (CI: − 0.26-0.04). There was no significant difference in expiratory waveforms (*p* = 0.2639).

**Conclusions:**

The AOA was non-inferior to the GOA for mask ventilation of patients with predictors of difficult mask ventilation and there was no significant difference in EtCO2 waveforms between the groups. These results were consistent in the subset of patients who were initially difficult to mask ventilate.

**Trial registration:**

ClinicalTrials.gov, NCT03144089, May 2017.

## Background

**Fig. 1:**
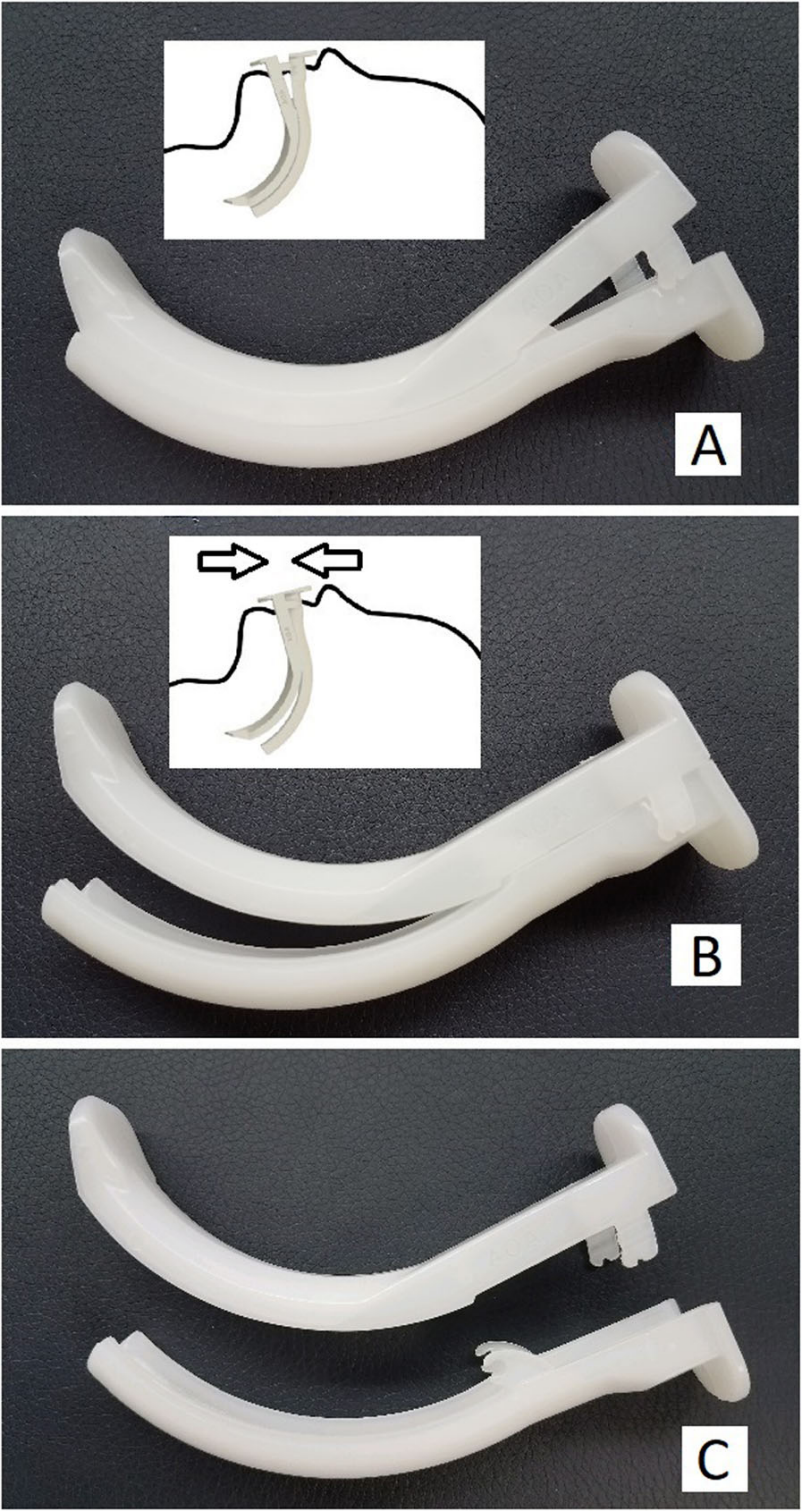
AOA in the closed (**a**), open (**b**), and disarticulated (**c**) conformations

Difficult mask ventilation is common in obese patients [1] and can result in morbidity and mortality [2]. The Guedel Oral Airway (GOA) is a commonly used device that acts as a static oropharyngeal stent and can result in greater airway patency and improved mask ventilation. The Articulated Oral Airway (AOA, Fig. 1) is a novel, commercially available, injection molded oral airway designed to facilitate flexible scope intubation via active displacement of the tongue (Fig. 1b) with the added benefit of easy post-intubation removal from the mouth via disarticulation (Fig. 1c).

Via this active tongue displacement, the AOA also has the potential advantage of increasing the cross-sectional area for mask ventilation. With evaluation of use for flexible scope intubations scheduled to follow, the primary aim of this study was to compare the GOA and AOA in regards to efficacy of mask ventilation and determine if the AOA, with its active tongue displacement, is non-inferior, thus demonstrating dual functionality of the device (flexible scope intubation and mask ventilation). We conducted a prospective, randomized trial comparing the efficacy of the GOA and AOA for mask ventilation of patients with factors predictive of difficult mask ventilation, with the hypothesis that the AOA would be non-inferior in regards to expiratory tidal volume. All non-tidal volume measure comparisons between GOA and AOA look for differences.

## Methods

This randomized clinical trial was approved by the University of Iowa Institutional Review Board and registered with ClinicalTrials.gov (NCT03144089, Principal investigator: Ron O. Abrons, Date of registration: 08/05/2017) prior to patient recruitment. This article adheres to applicable EQUATOR (Enhancing the QUAlity and Transparency Of health Research) Network guidelines. The study principle investigator (R.A.) or one of three research assistants reviewed the risks and benefits of the study with each patient, and written informed consent was obtained from all participants.

Study participants were patients 18 years of age or older with ≥2 predictors of difficult mask ventilation (MV), as listed below, who were scheduled to undergo elective surgery under general anesthesia including post-induction MV following administration of long-acting neuromuscular blockade. Patients were excluded if 1) emergency airway protection was needed, 2) there was a known history of failed MV or oropharyngeal anatomic abnormalities, 3) they were pregnant; 4) they were anticoagulated (beyond daily aspirin), 5) they were incarcerated, 6) they were unable to sign their own consent, 7) they needed inhalational induction, or 8) the distance from their maxillary incisors to the angle of the mandible was < 11 cm.

Predictors of difficult MV [3]

i. Age > 55 years
ii. Body mass index (BMI) > 30 kg/m^2^
iii. Presence of a beard
iv. Lack of teeth
v. History of snoring

Prior to the day of surgery, the operative scheduled was screened and those who met the above criteria were recruited on the day of surgery in the preoperative waiting area or inpatient hospital rooms. All participants were mask ventilated via both devices in random order. The order of the devices was randomized to account for the possibility of easier MV with the second device due to more profound paralysis [4] or more difficult MV with the second device due to provider fatigue [5]. Randomization was accomplished using a permuted block design with variable block sizes of two, four, and six and sealed opaque envelopes.

Each anesthesia team consisted of the patient’s staff anesthesiologist, the research anesthesiologist, a research assistant trained in appropriate placement of both the Guedel and Articulating Oral Airways, and a Certified Registered Nurse Anesthetist (CRNA). The staff anesthesiologist administered all medications while airway management was performed by the CRNA.

Pre-operatively, the staff anesthesiologist performed an examination noting age, body mass index (BMI), Mallampati grade [1–4], mouth opening (in centimeters, from the lower central incisor/gingiva to the upper central incisor/gingiva), neck extension (full, limited, or none), the presence of retrognathia (none, mild, or significant), the presence or absence of a beard, patient dentition, any apparent oropharyngeal anatomical abnormalities (“No” or “Yes,” with description), history of nightly snoring, and any diagnosis of sleep apnea with CPAP/BiPap settings.

Study equipment included a 100 mm GOA, a large AOA, an adult large anesthesia mask, two tongue depressors, a 28 Fr nasopharyngeal airway, water-soluble lubricant, an Entropy™ monitor, a pump for delivery of propofol, and a secure video recording device. To decrease intra-provider variability, breaths were delivered via the ventilator which was standardized to pressure control mode at 15 cm H_2_O with a rate of 10 breaths/minute and inspiratory:expiratory (I:E) ratio of 1:3 without positive end-expiratory pressure (PEEP), and oxygen flows of 10 l. To ensure blinding of the CRNA, the anesthesia mask was opacified with cloth tape and both oral airways were kept out of their sight at all times.

All patients were placed in supine position, and Entropy™ and quantitative train-of-four (TOF) monitors were applied in addition to American Society of Anesthesiologists (ASA) standard monitors. Preoxygenation was achieved with 100% O_2_ through the opacified face mask for ≥3 min or until end tidal O_2_ (EtO_2_) concentration was > 90%. Following pre-anesthetic time-out, general anesthesia was induced with intravenous fentanyl 50–100 mcg, lidocaine 1 mg·kg^− 1^ (up to 100 mg), and 1–3 mg·kg^− 1^ propofol. During MV anesthesia was maintained with an intravenous propofol infusion at 100 mcg/kg/min. After loss of the lid reflex, a baseline quantitative TOF ratio was obtained. A series of predetermined interventions were performed to characterize the difficulty of MV prior to administration of muscle relaxant and patient randomization. Successful MV was defined as that which resulted in the presence of end tidal CO_2_ (EtCO_2_) on the capnograph for multiple breaths. Initially, one-handed bag-MV, without an oral airway, was attempted by the CRNA utilizing an “EC grip” [6]. If one-handed MV without an oral airway was insufficient to return any EtCO_2_, a “two-handed jaw-thrust” technique [7, 8], without an oral airway, was utilized. If the two-handed technique was insufficient to return any EtCO_2_, a lubricated 28 Fr nasopharyngeal airway was placed and the two-handed technique restarted. If the two-handed MV with the 28 Fr nasopharyngeal airway did not return any EtCO_2_, the randomization protocol was initiated and the first randomized oral airway was inserted in addition to the nasopharyngeal airway. If there was no return of EtCO_2_ within five breaths with the first randomized device, it was removed and the device randomized to be second was placed. If, thereafter, bag mask ventilation was deemed impossible (no EtCO_2_ within five breaths with either oral airway), then the patient was immediately removed from the study protocol and the ASA Difficult Airway Algorithm was followed.

After confirmation of EtCO_2_, rocuronium 0.5–0.6 mg·kg^− 1^ (up to 50 mg) was administered intravenously and the research assistant opened the randomization envelope. Bag mask ventilation was continued until the TOF ratio was < 30% and the Entropy™ reading was < 50, with the administration of additional propofol or rocuronium as indicated to meet these goals.

When the above endpoints were achieved, the CRNA was asked to turn away so as to be blinded to oral airway placement order and the first randomized oral airway was placed. All oral airway placements were performed by either the research physician or research student (trained in proper oral airway placement and under the guidance of the research physician) for consistency. For placement of both oral airways, a tongue depressor was introduced into the oral cavity and the tongue displaced caudally. The devices were then inserted (the AOA in the “closed” conformation, Fig. 1a) with the concavity facing cephalad and then turned 180 degrees while advancing the proximal bevel to the level of the teeth. Once placed in the oropharynx, the AOA was then changed into the “open” conformation by bringing the proximal ends together (Fig. 1b). The opacified mask was then placed back on the patient and the CRNA resumed control of the mask using the two-handed jaw-thrust technique. The research physician ensured consistent mask ventilation technique between devices and between providers.

The ventilator was then turned on and video of the patient’s spirometry (inspired and expired tidal volumes to asses for leak) and EtCO_2_ waveforms were recorded from the anesthetic monitor. After 10 breaths, the CRNA stopped MV and again turned away from the patient. The first oral airway was removed by the research assistant (AOAs disarticulated prior to removal, Fig. 1c) and rotated in front of the video recorder to document the presence of any blood. The second oral airway was then placed by the research assistant, the opacified mask placed back on the patient, and MV was again undertaken by the CRNA. After the completion of an additional 10 ventilator-delivered breaths, the second oral airway was removed and the anesthetic continued per the primary team. If EtCO_2_ was not achieved after five breaths with the first oral airway, that device was removed and the second oral airway was placed. If EtCO_2_ was not achieved after five breaths with the second device, the protocol was aborted and the ASA Difficult Airway Algorithm was followed.

The research assistant noted the ease of initial MV (without oral airway, per CRNA report) and ease of oral airway placement (Easy < 5 s, Moderate 5–10 s, Difficult > 10 s to place). Approximately 35–45 min after the patient’s arrival in the post-anesthesia care unit, the research assistant documented any signs of oropharyngeal trauma and the patient’s subjective degree of sore throat (from 0 to 10).

The video recordings of the anesthetic monitor and post-removal oral airway were evaluated at a later date by one of two blinded reviewers (I.S. and K.U.) who were not involved in the reviewed patient’s care and not present in the room during the study period. Each video was cut into three separate videos: Device 1 (monitor only), Device 2 (monitor only), and Bleeding Check (first oral airway only). One of the above reviewers evaluated the Bleeding check videos and documented the presence or absence of blood on the first oral airway after removal. Separately, to ensure reviewer blinding to the placed oral airway, the other reviewer evaluated the Device 1 and Device 2 (monitor only) videos, recording the following data for breaths 6–10: inspiratory tidal volumes, expiratory tidal volumes, and EtCO_2_ waveform [V1 (waveform showing upslope, plateau and downslope phases), V2 (upslope and downslope, without plateau, phases), or V3 (no deviation from baseline) [9]].

The primary outcome of this study was efficacy of MV, as reflected by the average expiratory tidal volumes per kilogram body weight of breaths 6–10 via the AOA vs GOA. Breaths 6–10 were analyzed to allow for settling of the devices and adjustment by the provider to ensure both safe ventilation and limited movement during data point breaths. Secondary outcomes were 1) efficacy of MV, as reflected by the average inspiratory tidal volumes per kilogram body weight of breaths 6–10 via the AOA and GOA; 2) efficacy of MV, as reflected by evaluation of EtCO_2_ waveform of breaths 6–10 via the AOA and GOA; and 3) oropharyngeal trauma, as reflected by the presence or absence of blood on the device that was inserted first.

### Statistical analysis

The study was powered to test the hypothesis that weight-adjusted expiratory tidal volume measurements for the AOA are non-inferior to the GOA using a paired t-test. The non-inferiority margin for the difference between these two devices (GOA – AOA) was set as 1 ml/kg. The authors acknowledge that no data exists regarding what constitutes a clinically significant decrement in mask ventilation tidal volume and that the 1 ml/kg value is an estimation of what this may be. Since there are no mask-specific tidal volume data available for the power calculation, we used the expiratory tidal volume data (GOA-AOA = 0.34 ± 1.16) obtained from the initial 10 subjects. When the sample size is 55, a paired t-test with a one-sided type I error rate of 0.05 will have 69.2% power assuming a true mean difference of 0.66 ml/kg and standard deviation (SD) of 1.16 ml/kg. As an aside, a post-hoc power analysis shows a sample of 55 would result in 96.9% power for the test of non-inferiority at a margin of 1 ml/kg, considerably higher than originally anticipated.

Statistical comparisons between devices on patient characteristics were made using Fisher’s exact test and the Wilcoxon rank sum test for categorical and continuous variables, respectively. The generalized linear mixed modeling (GLMM) framework was used to compare the weight-standardized inspiratory/expiratory tidal volumes between the GOA and AOA (normal distribution). We also used GLMMs to assess if the proportion of abnormal waveforms differed between the two devices while controlling for first device used (Bernoulli distribution). The outcome of blood on the first-removed device was tested using Fisher’s exact test. Weight-standardized tidal volume mean differences between GOA and AOA will be reported along with their 95% confidence intervals to assess the non-inferiority hypothesis. All other measures were tested for differences between the devices. Comparisons with *p*-values < 0.05 are considered significant. Analyses were performed using SAS 9.4 (Cary, NC).

## Results

**Fig. 2:**
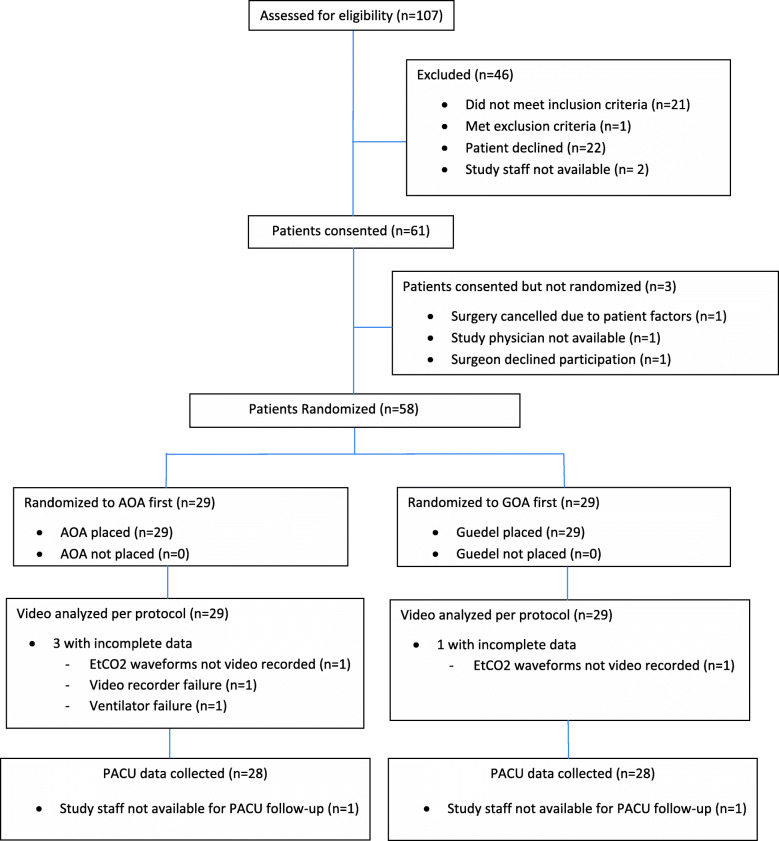
CONSORT flow chart

A total of 58 patients were consented and randomized (GOA placed first *n* = 29; AOA placed first n = 29) between 11 July 2017 and 25 February 2019. Two anesthesiologists and 33 CNRAs participated in patient care for this study. All oral airways were placed by one of two members of the research team (one physician and one senior medical student). There were no protocol violations, but complete data could not be collected for four patients due to failures in spirometry, capnography, the ventilator circuit, and video collection, respectively. As tidal volume and waveform data were analyzed independently, when tidal volume data was present, but waveform data absent, the tidal volume was still analyzed and vice-versa. The number of patients at each study phase and details of data loss are presented in the CONSORT flow chart (Fig. 2).

Patient demographics, preoperative airway morphology, and ease of MV without an oral airway are summarized in Table 1 stratified by randomized order of devices.

**Table 1:** Demographics, pre-operative airway examination measures, and intubation variables

Variable	Guedel placed first (***n*** = 29)	AOA placed first (n = 29)
**Sex; male**	**16 (55.2%)**	**20 (69.0%)**
Age; years	60 (53–65)	64 (58–68)
**Body mass index**	**34.8 (31.0–39.0)**	**36.1 (33.0–40.0)**
Presence of facial hair	11 (37.9%)	13 (44.8%)
**Presence of teeth**	**15 (51.7%)**	**17 (58.6%)**
History of Snoring	26 (89.7%)	28 (96.6%)
**Formal diagnosis of obstructive sleep apnea**	**14 (48.3%)**	**14 (48.3%)**
Neck extension		
Full	29 (100%)	26 (89.7%)
Limited	0 (0%)	3 (10.3%)
**Mouth opening; cm**	**4.5 (4.0–5.0)**	**5.0 (4.0–5.5)**
Presence of retrognathia	4 (13.8%)	7 (24.1%)
**Distance from maxillary incisors to angle of mandible; cm**	**11.5 (11.0–12.0)**	**11.5 (11.0–12.0)**
Nasal/Oral anatomical abnormality	0 (0%)	0 (0%)
**Mallampati Grade**		
1	**5 (17.2%)**	**7 (24.1%)**
2	**18 (62.1%)**	**12 (41.4%)**
3	**4 (13.8%)**	**9 (31.0%)**
4	**2 (6.9%)**	**1 (3.5%)**
Easy one-handed mask ventilation *without* oral airway	21 (72.4%)	22 (78.6%)

The two groups did not differ in terms of age, BMI, sex, mouth opening, Mallampati grade, degree of retrognathia, distance from maxillary incisors to the angle of the mandible, history of snoring, formal diagnosis of obstructive sleep apnea, or presence or absence of oral or nasal anatomical abnormalities, facial hair, or teeth. Mask ventilation was established in all but two patients (one due to patient factors and one due to ventilator circuit failure), both of whom were successfully intubated per protocol without drop in oxygen saturation < 94%. One patient could only be mask ventilated when the GOA was exchanged for the AOA. There were no incidents of dental damage or study-related adverse events. One patient was noted to have a small palatal excoriation (1 mm) postoperatively, but it is unclear if this was due to oral airway or endotracheal tube placement.

The primary outcome of weight-standardized expiratory tidal volumes, controlling for device randomization order, showed a mean difference (GOA – AOA) of 0.452 ml/kg (95% confidence interval (CI): 0.335 to 0.568), which was within the margin of non-inferiority (1 ml/kg, Table 2).

**Table 2:** Comparison of weight-standardized expiratory tidal volumes controlling for device randomization order

Variable	Guedel vs. AOA	*p*-value
Expiratory tidal volume	Mean diff = 0.452 (ml/kg) CI = (0.335 to 0.568)	
Inspiratory tidal volume	Mean diff = −0.109 (ml/kg) CI = (− 0.261 to 0.044)	
Ease of placement	^a^Odds ratio = 0.136 CI = (0.007 to 2.684)	0.24
Normal expiratory waveform	Odds ratio = 1.772 CI = (0.650 to 4.832)	0.26
Blood on 1st device (Guedel 1st vs. 2nd)	Odds ratio = 7.793 CI = (0.384 to 157.972)	0.24

^a^ = corrected odds ratio for 0-cell count

A similar non-inferiority was seen in inspiratory volumes (GOA – AOA = − 0.109 ml/kg; CI: − 0.261 to 0.044). There were no significant differences in ease of placement (*p* = 0.24) or expiratory CO_2_ waveforms (*p* = 0.26) between the devices. The GOA-first group had three subjects with blood present on the device after removal, as compared to none with the AOA, but this was not significant (p = 0.24). Of note, while volumes are measured to the nearest milliliter, when adjusted for weight (kg) the scale shrinks and more precise values (fractions of ml/kg) are needed than for an unadjusted comparison.

When considering the subset of 14 patients who were not initially easy to mask ventilate (unsuccessful one-handed MV without adjuncts), comparisons for weight-standardized expiratory tidal volumes controlling for device randomization order showed a mean difference between the GOA and AOA of 0.397 ml/kg (CI: 0.134 to 0.660). Consistent with the total study population, this was within the margin of non-inferiority (1 ml/kg, Table 3).

**Table 3:** Comparison of weight-standardized expiratory tidal volumes in patients not initially easy to mask ventilate

Variable	Guedel vs. AOA	*p*-value
Expiratory tidal volume	Mean diff = 0.397 (ml/kg) CI = (0.134 to 0.660)	
Inspiratory tidal volume	Mean diff = − 0.041 (ml/kg) CI = (− 0.321 to 0.240)	
Ease of placement	^a^Odds ratio = 0.312 CI = (0.012 to 8.285)	> 0.999
Normal expiratory waveform	Odds ratio = 1.536 CI = (0.238 to 9.926)	0.65
Blood on 1st device (Guedel 1st vs. 2nd)	^a^Odds ratio = 5.769 CI = (0.232 to 143.371)	0.47

^a^ = corrected odds ratio for 0-cell count

For comparison of inspiratory measurements, the same non-inferiority was seen between the two devices (GOA – AOA = − 0.041; CI: − 0.321 to 0.240). The test for waveforms also failed to find a significant difference in odds of V1 waveforms for either device (*p* = 0.65). No significant differences were seen for blood on first device after removal (*p* = 0.47).

## Discussion

This study observed the Articulated Oral Airway to be non-inferior to the Guedel Oral Airway during mask ventilation of patients with factors predictive of difficult mask ventilation. This was true for the non-inferiority primary outcome measure (expiratory tidal volume), as well as all other tests of superiority. These findings were also present in patients who were initially difficult to mask ventilate (unsuccessful MV with one-handed EC grip and no adjuncts).

The concept of active tongue displacement during MV is not new, with the cuffed oropharyngeal airway (COPA) being well studied [10–14]. The AOA likely works in a similar fashion, with the benefit of a larger lumen (which can accommodate an 8.5 subglottic suction endotracheal tube) and the ability to disarticulate the AOA around the endotracheal tube after successful intubation. Although the utility of the AOA for flexible scope intubation will be studied in the near future, this study demonstrates its non-inferiority to the GOA for the fundamental task of MV.

A limitation of this study is that the “impossible to mask ventilate” scenario is uncommon [1, 2] and the benefit of any device in that situation is unlikely to be delineated in a prospective manner. In this study only one patient could not be mask-ventilated with the combined use of oral airway, nasopharyngeal airway, and two-handed MV. An additional patient could only be mask ventilated when the GOA was replaced with the AOA, but this observation is inconclusive. Another limitation of the study is that difficulty in MV is multifactorial, with patient, provider, and equipment factors all playing roles. Although patients were screened for size likely to accommodate a 100 mm GOA and large AOA, sizing guidelines are only estimates and external anatomy does not necessarily reflect oropharyngeal space. In addition, as efficacy and endurance of MV likely also vary among providers, inclusion of multiple CRNAs allows for the possibility of inter-provider variances.

## Conclusions

The Articulating Oral airway is non-inferior to the Guedel Oral Airway for mask ventilation of patients with predictors for difficult mask ventilation. Though it was designed as an adjunct for flexible scope intubation, these results show the AOA is also efficacious for mask ventilation.

## Data Availability

The datasets used and/or analyzed during the current study are available from the corresponding author on reasonable request. All others authors (I. Sheffield and P. Ten Eyck) have no conflicts.

## Abbreviations

AOA: Articulated Oral Airway
GOA: Guedel oral airway
EQUATOR: (Enhancing the QUAlity and Transparency Of health Research)
SD: Standard deviation
MV: Mask Ventilation
BMI: Body mass index
CRNA: Certified Registered Nurse Anesthetist
CPAP: Continuous positive airway pressure
BiPap: Bilevel positive airway pressure
PEEP: Positive end-expiratory pressure
TOF: Train-of-four
ASA: American Society of Anesthesiologists
EtO2: End tidal O_2_
EtCO2: End tidal CO_2_
GLMM: Generalized linear mixed modeling
COPA: Cuffed oropharyngeal airway

## References

[1] Kheterpal S, Han R, Tremper KK, Shanks A, Tait AR, O’Reilly M, Ludwig TA (2006). Incidence and predictors of difficult and impossible mask ventilation. Anesthesiology.

[2] Kheterpal S, Martin L, Shanks AM, Tremper KK (2009). Prediction and outcomes of impossible mask ventilation: a review of 50,000 anesthetics. Anesthesiology.

[3] Langeron O, Masso E, Huraux C, Guggiari M, Bianchi A, Coriat P, Riou B (2000). Prediction of difficult mask ventilation. Anesthesiology.

[4] Joffe AM, Ramaiah R, Donahue E, Galgon RE, Thilen SR, Spiekerman CF, Bhananker SM (2015). Ventilation by mask before and after the administration of neuromuscular blockade: a pragmatic non-inferiority trial. BMC Anesthesiol.

[5] Khoury A, De Luca A, Sall FS, Pazart L, Capellier G (2015). Performance of manual ventilation: how to define its efficiency in bench studies? A review of the literature. Anaesthesia.

[6] Ortega R, Mehio AK, Woo A, Hafez DH (2007). Videos in clinical medicine. Positive-pressure ventilation with a face mask and a bag-valve device. N Engl J Med.

[7] Safar P, Escarraga LA, Elam JO (1958). A comparison of the mouth-to-mouth and mouth-to-airway methods of artificial respiration with the chest-pressure arm-lift methods. N Engl J Med.

[8] Joffe AM, Hetzel S, Liew EC (2010). A two-handed jaw-thrust technique is superior to the one-handed "EC-clamp" technique for mask ventilation in the apneic unconscious person. Anesthesiology.

[9] Japanese Society of A (2014). JSA airway management guideline 2014: to improve the safety of induction of anesthesia. J Anesth.

[10] Koga K, Sata T, Kaku M, Takamoto K, Shigematsu A (2001). Comparison of no airway device, the Guedel-type airway and the cuffed oropharyngeal airway with mask ventilation during manual in-line stabilization. J Clin Anesth.

[11] Rees SG, Gabbott DA (1999). Use of the cuffed oropharyngeal airway for manual ventilation by nonanaesthetists. Anaesthesia.

[12] Asai T, Koga K, Jones RM, Stacey M, Latto IP, Vaughan RS (1998). The cuffed oropharyngeal airway. Its clinical use in 100 patients. Anaesthesia.

[13] Asai T, Koga K, Stacey MR (1997). Use of the cuffed oropharyngeal airway after difficult ventilation through a facemask. Anaesthesia.

[14] Uezono S, Goto T, Nakata Y, Ichinose F, Niimi Y, Morita S (1998). The cuffed oropharyngeal airway, a novel adjunct to the management of difficult airways. Anesthesiology.

